# The significance of chemical reaction, thermal buoyancy, and external heat source to optimization of heat transfer across the dynamics of Maxwell nanofluid via stretched surface

**DOI:** 10.1038/s41598-024-55419-5

**Published:** 2024-03-11

**Authors:** Bilal Ahmad, Bagh Ali, Abdul Bariq, Muhammad Ozair Ahmed, Syed Asif Ali Shah, Muhammad Idrees, Adham E. Ragab

**Affiliations:** 1 Department of Mathematics and Statistics, The University of Lahore, Lahore, 54000 Pakistan; 2 School of Mechanical Engineering and Automation, Harbin Institute of Technology, Shenzhen, 518055 China; 3 Department of Mathematics, Laghman University, Mehtarlam City, 2701 Laghman Afghanistan; 4 Department of Industrial Engineering, College of Engineering, King Saud University, P.O. Box 800, Riyadh, 11421 Saudi Arabia

**Keywords:** Nanofluid, MHD, Mixed convection, Activation energy, Shrinking medium, Maxwell fluid, Engineering, Mathematics and computing, Engineering, Mathematics and computing

## Abstract

Energy loss during the transportation of energy is the main concern of researchers and industrialists. The primary cause of heat exchange gadget inefficiency during transportation was applied to traditional fluids with weak heat transfer characteristics. Instead, thermal devices worked much better when the fluids were changed to nanofluids that had good thermal transfer properties. A diverse range of nanoparticles were implemented on account of their elevated thermal conductivity. This research addresses the significance of MHD Maxwell nanofluid for heat transfer flow. The flow model comprised continuity, momentum, energy transport, and concentration equations in the form of PDEs. The developed model was converted into ODEs by using workable similarities. Numerical simulations in the MATLAB environment were employed to find the outcomes of velocity, thermal transportation, and concentration profiles. The effects of many parameters, such as Hartman, Deborah, buoyancy, the intensity of an external heat source, chemical reactions, and many others, were also evaluated. The presence of nanoparticles enhances temperature conduction. Also, the findings are compared with previously published research. In addition, the Nusselt number and skin friction increase as the variables associated with the Hartman number and buoyancy parameter grow. The respective transfer rates of heat are 28.26$\%$ and 38.19$\%$ respectively. As a result, the rate of heat transmission increased by 14.23$\%$. The velocity profiles enhanced while temperature profiles declined for higher values of the Maxwell fluid parameter. As the external heat source increases, the temperature profile rises. Conversely, buoyancy parameters increase as it descends. This type of problem is applicable in many fields such as heat exchangers, cooling of electronic devices, and automotive cooling systems.

## Introduction

In recent decades, there has been significant interest in the production and application of microdevices. Microtechnology mechanisms offer numerous benefits, including fabricating microdevices (e.g., microsensors, microvalves, and micropumps) in minute dimensions and with significant efficiency. The magnification of efficiency and keeping them cool during work is an apprehension. There has been tremendous research on the flow and transfer of nanofluids, and it is still an active research area. Nanofluids play a very important role in heat and mass transfer phenomena.^1^ is one of the pioneers of this field who introduced nanoparticles in fluid flow after remarkable changes happened in heat mass transformation and fluid flow. Then^2^ investigated the Tiwari-Das model with the Dufour Sorrot effect over $Al_3$-water nanofluid flow over the needle.^3^ worked the magnetic field, thermal radiation, and convection effects in a square body filled with $TiO_2$.^4,5^ discussed the stagnation point flow for the Tiwari and Das model over a stretching sheet with a slip effect.^6^ investigated the impact of MHD effects shape of the nanoparticles and thermal radiations through the sheet.

There are many applications of magnetic hydrodynamics, including liquid flow and temperature transport, peristaltic flow, ship propulsion, metallurgy, crystal growth, fusion reactors, etc. Researchers use magnetic nanoparticles. At present, there are a variety of industries that use magnetic fields. The use of MHD nanofluids in biological imaging and several other fields is wide-ranging.^7,8^ used the FEM method to look at the flow of MHD nanofluid and see how melting affected Cattaneo-Christov and thermal radiation.^9^ discussed the MHD nanofluid flow between two cylinders in a theoretical analysis. The results showed that while the temperature profile decreased with rising Hartmann numbers and radiation criteria, it improved with the ascent of Reynolds and Eckert numbers. An octagon container with fins was used in the work by^10^ to analyse the entropy of buoyancy-driven magnetohydrodynamic hybrid nanofluid flow. The results of the investigation showed that while the magnetic number decreased with nanoparticle concentrations, entropy rose.^11^ discussed ferrofluid movement across a rotating disc in the presence of a strongly fluctuating magnetic field. The findings showed that a higher field frequency raised the temperature, while a smaller nanoparticle diameter decreased heat transmission. The stability analysis of an MHD hybrid nanofluid flow with a quadratic speed over a stretching sheet was talked about in^12^. The initial solution’s positive minimum eigenvalue was shown by stability analysis, which adequately defined a steady and achievable flow.^13^ investigated the MHD fluid flow with multi-slip effects over the permeable sheet. The thermodynamic properties of hybrid and traditional nanofluid flows across a curvy, sliding porous surface were studied by^14^. Additionally, it was believed that the surface is wrapped inside a circular sphere. Many other researchers also work on MHD fluid flow, like^15–18^.

These days, fluid flow under the influence of mixed convection appeals to researchers. Mixed convection phenomena are widely employed in the construction of industrial techniques, e.g., cooling of electronic devices, heat exchanging from nuclear reactors, biomedical sciences, and many other technological applications.^19^ investigated heat transfer through the flow of a nanofluid in the presence of a magnetic effect through a rotating system. Because of the Lorentz forces, it was discovered that the Nusselt number fell as the magnetic parameter increased.^20^,? elaborated on the nanofluid Brownian motion and thermophoresis through a rotating stretching sheet.^21^ explored the fluctuation in temperature profiles of MHD nanofluid flow over the stretching velocity of CU-water over the rotating frame. Enhancing the combined convection and magnetic parameters produces a delay in the boundary layer separation.^22^ discussed the bouncy and effects of radiation for MHD micropolar nanofluid flow over rotating pours stretching sheet a numerical investigation. The velocity profile decreased as the magnetic parameter M increased. Conversely, when the magnetic parameter M grew, so did the micro-rotation. The movement of a MWCNT-Fe3O4-water hybrid nanofluid across a micro-wavy conduit was investigated by^23^.

The stretching/shrinking phenomena are one of the key characteristics of nanofluid flow. The pioneer of this study is^24^, who introduced the phenomena. This concept was later used by many scientists to achieve further accomplishments like Hayat et al.^25^ discussed the heat transportation with stretchable sheets for MHD flow.^26^ examined the relevance of temperature gradient-induced fluctuation in the diameter, mass flow, and dissipation of energy in the fluid’s convection radiation motion, the heat source, and the Darcy-Forchheimer model across a cylinder. Because of the density spectrum, the energy fluxion was noticeably larger in magnitude due to an enlarged nanoparticle’s diameter, which also dramatically reduced the temperature profile across the domain.^27^ studied the boundary layer flow of micro-polar nanofluid over a stretching sheet with thermal radiations.^28^ studied the slip flow of Power-Eyring nanofluid flow with nonsteady MHD over a permeable stretching sheet.^29^ elaborated FEM simulations for unsteady MHD Maxwell nanofluid flow with effects of thermo diffusion and chemical reactions along stretching sheets.

The writer behind this work intends to study the computational evaluation to enhance the heat transfer maxwell nanofluid flows by using a two-dimensional porous sheet by external heat source with activation energy and mixed convection, as there has been no research on this subject to the best of our knowledge. The findings of this study expand on the impact of the velocity profiles, temperature profiles, and concentration profiles of nanofluids. The governing equations are transformed into dimensionless equations with the dimensionless parameters given. The final nonlinear governing equations were numerically solved using MATLAB. The features of parameters for the velocity profile, temperature profile, concentration profile, and micromotile profile are addressed tabularly and visually, and they are also compared to a few previous findings. This article’s results and comments may be useful.

### Research questions

The following scientific research issues are addressed in this chapter:

1. What is the effect of Lorentz forces, bouncy forces, and the velocity ratio parameter on the velocity profile?
2. How heat transported when the magnetic field, the bouncy characteristic, heat conduction, and Brownian motion are present?
3. What is the effect on nanoparticle concentration under the influence of the magnetic field, bouncy parameter, thermophoresis, and Brownian motion?
4. How skin friction, Nustle number, and Sherwood number change with the effect of the magnetic field, activation energy, and mixed convection parameter?

## Purposed model

**Figure 1:**
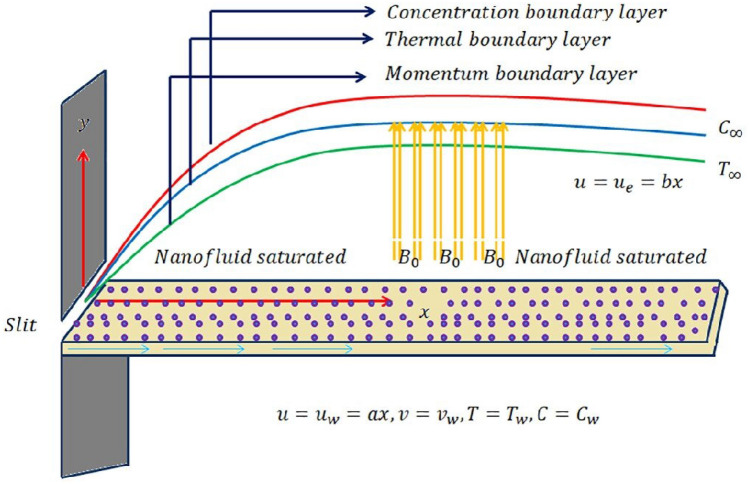
Geometry of purposed model.

A two-dimensional laminar Maxwell nanofluid flow with steady boundary conditions was evaluated under the influence of activation energy, mixed convection, and stretching effects, about Fig. 1. A constant magnetic field is applied perpendicularly to the flow surfaces to balance the boundary layers with a permanent heat source. Consider that $u_w(x) = ax, a>0$, and $u_e(x) = bx, b>0$ are the stretching velocity and stream velocity respectively. Now the governing equations for fluid flow, heat transportation, and consecration of nanoparticles are given below^30–32^

1 $$\begin{aligned}{} & {} \frac{\partial u}{\partial x}+\frac{\partial v}{\partial y}= 0, \end{aligned}$$

2 $$\begin{aligned}{} & {} u\frac{\partial u}{\partial x}+v\frac{\partial u}{\partial y}= u_e\frac{du_e}{dx}+v\frac{\partial ^2u}{\partial y^2}-\lambda ^{*}\nonumber \\{} & {} \Big [{u}^{2}\frac{{\partial }^{2}u}{\partial {x}^{2}}+2uv\frac{{\partial }^{2}u}{\partial x\partial y}+{v}^{2}\frac{{\partial }^{2}u}{\partial {y}^{2}}\Big ]\nonumber \\{} & {} -\frac{\sigma ^{*}}{\rho }B_o^2u + g\beta _{t}(T - T_\infty )+g\beta _{c}(C - C_\infty ), \end{aligned}$$

3 $$\begin{aligned}{} & {} u\frac{\partial T}{\partial x}+v\frac{\partial T}{\partial y}=\alpha \frac{\partial ^2T}{\partial y^2}+\frac{Q}{\rho C_p}(T - T_\infty )+\tau \Big [D_B \frac{\partial T}{\partial y} \frac{\partial C}{\partial y}+\frac{D_T}{T_\infty }\left( \frac{\partial T}{\partial y}\right) ^2\Big ], \end{aligned}$$

4 $$\begin{aligned}{} & {} u\frac{\partial C}{\partial x}+v\frac{\partial C}{\partial y}=\frac{D_T}{T_\infty }\frac{\partial ^2 T}{\partial y^2}-K_r\left( \frac{T}{T_\infty }\right) ^n e^\frac{-Ea}{kT}(C-C_\infty )+D_B\frac{\partial ^2 C}{\partial y^2}. \end{aligned}$$

Appropriate boundary conditions for Eqs. (1–4) are

5 $$\begin{aligned} u = u_w,\ v=0,\ T=T_w,\ C=C_w,\ {\textbf {when}}\ y=0, \end{aligned}$$

6 $$\begin{aligned} u \longrightarrow 0,\ T\longrightarrow T_\infty ,\ C\longrightarrow C_\infty ,\ {\textbf {when}}\ y\longrightarrow \infty . \end{aligned}$$

    Here the velocity along the *x*-axis is *u* and the *y*-axis is *v* respectively. In the above equations, a few terms appeared e.g. $B_o$ magnetic field, $\sigma ^{*}$ electric conductivity, the thermal expansion is $\beta _{t}$, the density of fluid is $\rho$, *T* is the ambient temperature, *Q* is heat source coefficient, the concentration expansion is $\beta _{c}$, $\alpha$ represents the thermal diffusion, $C_p$ is specific heat coefficient, $D_B$ is brownian diffusion coefficient, $D_T$ is thermophrosis diffusion coefficient, $\tau$ is specific heat of nanoparticles, $E_a$ is activation energy, *k* is Boltzmann constant.

In order to advance the investigation, We’ll apply the similarity transformation below. Considering^30^.

7 $$\begin{aligned} \eta =\sqrt{\frac{u_w}{vx}}y,\ \theta (\eta )=\frac{T-T_\infty }{T_w-T_\infty }, u=bxf'(\eta ), \ v=-\sqrt{bv}f(\eta ), \phi (\eta )=\frac{C-C_\infty }{C_w-C_\infty }. \end{aligned}$$

The Eq. (1) is satisfied identically by employing transformation, from Eqs. (2–4) converted into nonlinear ordinary differential equations as

8 $$\begin{aligned} f''' + ff'' - f'^2 + M(1-f')+1+\lambda \theta + Nr\phi +\beta \Big [2f''f'f+f^2+f'''\Big ] = 0, \end{aligned}$$

9 $$\begin{aligned} \frac{1}{Pr} \theta ''+f\theta ' + Q_s \theta +Nb \theta ' \phi '+Nt\theta '^2 = 0, \end{aligned}$$

10 $$\begin{aligned} \phi ''+Sc f\phi '- \sigma (1 + \theta \delta ).exp\left( \frac{-E}{\theta \delta +1}\right) ^n Sc \phi +\frac{Nt}{Nb}\theta ''=0. \end{aligned}$$

The boundary conditions also converted

11 $$\begin{aligned} f(\eta )=0,\ f'(\eta )=\epsilon ,\ \theta (\eta )=1,\ \phi (\eta )=1\ {\textbf {at}}\ \eta =0, \end{aligned}$$

12 $$\begin{aligned} f'(\eta ) \longrightarrow 1,\ \theta (\eta ) \longrightarrow 0,\ \phi (\eta )\longrightarrow 0\ {\textbf {when}}\ \eta \longrightarrow \infty \end{aligned}$$

In the proposed model, which is mentioned in equations 8-10, a few dimensionless values appear after leveraging the similarities mentioned above and simplifying the above model. Here, $M = \frac{\sigma B^2 }{\rho b}$ known as the Hartman number, $P_r = \frac{\nu }{\alpha }$, known as the Prandtl number, $Nr = \frac{Gr}{Re^2}$ known as the free convection parameter, $Gr=\frac{g\beta _t (T-T_\infty )x^3}{\nu ^2}$ is the Grashof number, $R_e=\frac{ux}{\nu }$ is Reynolds number^33^, $\epsilon =\frac{a}{b}$ indicates velocity ratio parameter, $Nb = \frac{\tau D_b(C_w-C_\infty )}{\nu }$ known as the value of Brownian motion, $\beta = b \lambda ^{*}$ denotes Maxwell fluid parameter, $\lambda = \frac{g\beta _t(T_w-T_{\infty })}{b^{2} }$ is thermal free convection parameter, $Q_s = \frac{Q}{\rho b C_p}$ is heat generation parameter, and and $Nt = \frac{\tau D_b(T_w-T_\infty )}{T_\infty \nu }$ is known as the value of thermophoresis.

The skin friction coefficient and Nusselt number are defined as

13 $$\begin{aligned} Cf = \frac{\mu }{\rho (ax)^2}\left( \frac{\partial u}{\partial y}\right) _{y=0} \end{aligned}$$

14 $$\begin{aligned} Nu = -\frac{x}{k_f(T-T_\infty )}\left( k_f\frac{\partial T}{\partial y}\right) _{y=0} \end{aligned}$$

By using the similarity transformations from Eq. (7) into Eqs. (13) and (14), we obtain

15 $$\begin{aligned} (Re_x)^{0.5} Cf = f''(0) \end{aligned}$$

16 $$\begin{aligned} (Re_x)^{-0.5} Nu = -\theta '(0) \end{aligned}$$

## Numerical scheme

The solution of nonlinear ODEs (8–10) has been required to find the solution of appeared physical quantities in the proposed model. The Runnga-kutta numerical method is applied to reduce the nonlinearity of the model. The solution has been found with the help of MATLAB. The higher order ODEs converted as follows.

$$\begin{aligned} S_1=f, S_2=f', S_3=f'', S_3'=f''',S_4=\theta , S_5 = \theta ', S_5' = \theta '', S_6 = \phi , S_6' = \phi ',\\ S_1'=S_2,\\ S_2'= S_3,\\ S_3' = \frac{1}{1+\beta }\left[ S_2^2 - S_1S_3 - M(1 - S_2) - 1- \lambda S_4 - 2\beta \left[ S_1S_2S_3 + S_1^2 \right] \right] ,\\ S_4' = S_5\\ S_5' = -P_r \left[ S_1S_5+Q_sS_4+NbS_5S_6+NtS_5^2 \right] ,\\ S_6'=\frac{1}{S_1}\left[ \sigma (1+S_4\delta )exp^ {\frac{-E}{S_4\delta +1}}ScS_6-\frac{Nt}{Nb}S_5 \right] . \end{aligned}$$

The boundary conditions are indeed modified similarly.

$$\begin{aligned} S_1 = 0,\ S_2=\epsilon ,\ \theta (\eta )=1,\ \phi (\eta )=1\ {\textbf {at}}\ \eta =0 \\ S_1 \longrightarrow 1,\ S_4 \longrightarrow 0,\ S_6 \longrightarrow 0\ {\textbf {when}}\ \eta \longrightarrow \infty \end{aligned}$$

## Model validation

We drew a comparison with the existing litterateur for validation of the proposed flow model. The most significant results have been compared in Tables 1 and 2 to previous research of^30,34^, and the validity of the results is proved for limited cases. The mathematical model uses the table and fixed, dimensionless numbers for parameters to look at what happens when thermal radiation, viscosity, and Arrhenius energy change. We have $\beta =1.0, \lambda =0.15, M=1.0, Pr=2.0, Sc=1.0, \sigma =0.50, \delta =0.3, m=0.5, E=0.3, Nb=0.15, Nt=0.21, Nr=0.2, \textrm{and} Rb=0.2$. These comparison tables show the validation of the results with existing literature comprehensively.

**Table 1:** Comparison of $f''(0)$ and $\theta '(0)$ with *Pr* under the controlled parameters.

Pr	^34^		^30^		New results	
	$-f''(0)$	$\theta '(0)$	$-f''(0)$	$\theta '(0)$	$-f''(0)$	$\theta '(0)$
1.0	1.6754	0.8708	1.6786	0.8717	1.67878	0.87181
2.0	1.6123	1.1151	1.6162	1.1157	1.61649	1.11573
3.0	1.5821	1.2839	1.5829	1.2864	1.58307	1.28649
4.0	1.5601	1.4215	1.5612	1.4221	1.56139	1.42218

**Table 2:** Comparison of $f''(0)$ and $\theta '(0)$ with *M* under the ontrolled parameters.

M	^34^		^30^		New results	
	$-f''(0)$	$\theta '(0)$	$-f''(0)$	$\theta (0)'$	$-f''(0)$	$\theta '(0)$
0.0	0.94095	0.57333	0.94096	0.57339	0.9409701	0.573452
1.0	1.06960	0.58860	1.06964	0.58866	1.0696521	0.588713
2.0	1.58290	0.61430	1.18340	0.59994	1.1834097	0.599982
4.0	2.22158	0.66730	1.38201	0.61869	1.3820189	0.618701

## Results and discussion

In this chapter, we investigated the magnetized Maxwell Nanofluid flow with activation energy, mixed convection, and heat source over the stretching sheet under the limiting condition with flowing fixed values: $\beta =1.0, \lambda =0.5, M=1.0, Pr=2.0, Sc=1.0, \sigma =0.5, \delta =0.3, n=0.5, E=0.3, Nb=0.15, Nt=0.21, Nr=0.2, Q_s = 0.2, \epsilon =0.2$. The impact of these parameters is discussed over the momentum profile, energy profile, and concentration profile of nanofluid.

**Figure 2:**
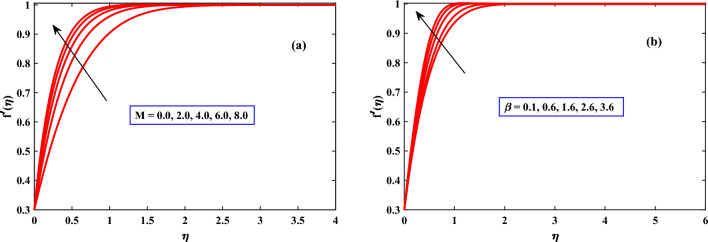
Behaviour of velocity under the influence of *M* and $\beta$.

**Figure 3:**
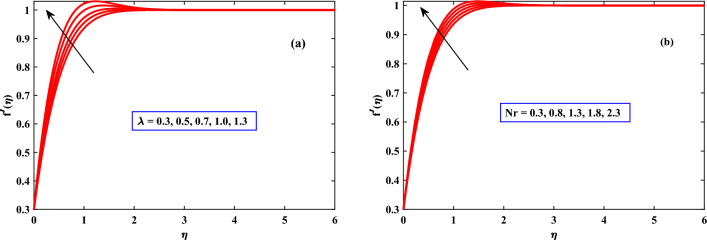
Behaviour of velocity under the influence of *Nr* and $\lambda$.

**Figure 4:**
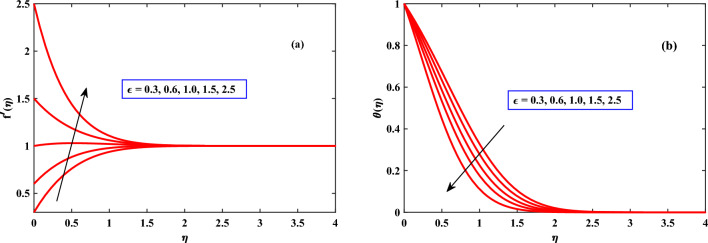
Behaviour of velocity and temperature under the influence of $\epsilon$.

In Figs. 2, 3 and 4 the behavior of the velocity profile is discussed for different parameters. The velocity profile shows dynamically increasing behavior due to the increased values of Harmat number *M*, Mexwell parameter $\beta$, stretching parameter$\epsilon$, buoyancy parameter $\lambda$, and density ratio *Nr* respectively. An increase in Hartmann Number in Fig. 2 can reduce the fluid’s boundary layer movement. Since the Lorentz force is created by the transversal magnetic field in electrically conductive fluids. Free stream speed, however, transcends the stretching surface speed when the magnetic parameter is countered with the velocity distribution^35,36^. When the Maxwell parameter $\beta$ (Deborah number) as shown in Fig. 2 increases, it indicates that elastic response dominates viscous response. Hence, the fluid can store and release elastic energy significantly. Due to its elasticity, the fluid is capable of accelerating and increasing its velocity more rapidly when subjected to external forces or disturbances. Figure 3 shows the impact of the density ratio *Nr* on the velocity profile. The velocity profile increases as the density ratio increases, indicating that the density ratio aids in the system’s stabilization. When the buoyancy parameter $\lambda$ through Fig. 3 increases, a difference in densities within the fluid and outside the fluid will take place fluid gets heated and becomes less dense, it tends to rise, while the cooler. As a result, the buoyancy forces become more pronounced and can drive more vigorous fluid motion. Thus, the fluid accelerates, and its velocity increases. When the stretching parameter $\epsilon$ increases the cross-sectional area of the flow geometry decreases, causing the fluid to accelerate to maintain a constant flow rate as indicated in Fig. 4. Thus, the fluid accelerates, and its velocity increases.

**Figure 5:**
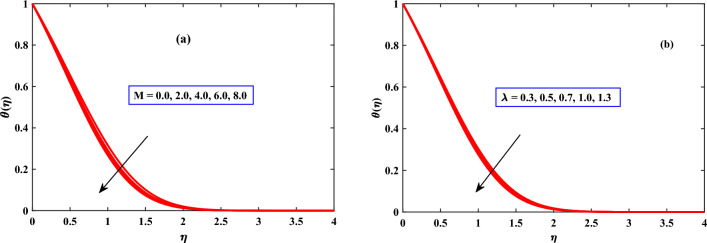
Behavior of temperature under the influence of $\lambda$ and *M*.

**Figure 6:**
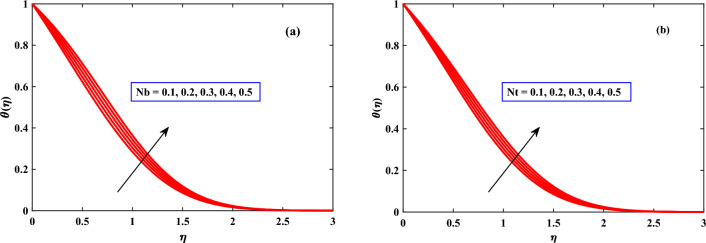
Behaviour of temperature under the influence of *Nb* and *Nt*.

**Figure 7:**
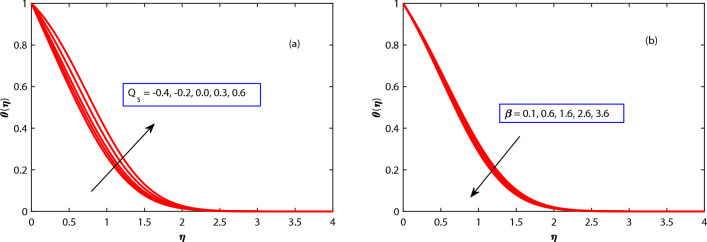
Behaviour of temperature under the influence of *Qs* and $\beta$.

In Figs. 4b, 5, 6 and 7 the behavior of the temperature profile is discussed for different parameters. The temperature profile shows dynamically increasing behavior for the boosted values of Brownian motion *Nb*, thermophoresis *Nt*, and heat sources *Qs* but for maxwell parameter $\beta$, Harmat number, stretching parameter $\epsilon$, and buoyancy parameter$\lambda$ showed diminished behavior respectively. When the stretching parameter $\epsilon$ is increased, the internal energy of the fluid is converted into work during the stretching process as shown in Fig. 4. The volume of the fluid grows while the pressure falls as it is stretched. The ideal gas law states that as a gas’s pressure lowers, so does its temperature. The temperature gradient in the fluid is raised as the thermophoresis *Nt* is increased as depicted in Fig. 6, creating a force that acts on the suspended particles. This force can cause particles to travel towards regions of higher temperature.

**Figure 8:**
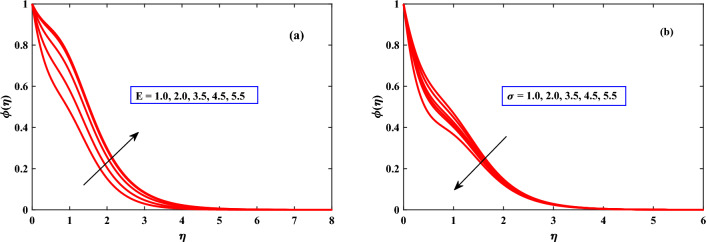
Behaviour of concentration under the influence of *E* and $\delta$.

In Fig. 8 , the actions that were taken on the concentration profile are discussed for different parameters. The concentration profile shows dynamically increasing behavior for the boosted values of activation energy *E* but reaction rate $\sigma$ showed diminished behavior.

**Figure 9:**
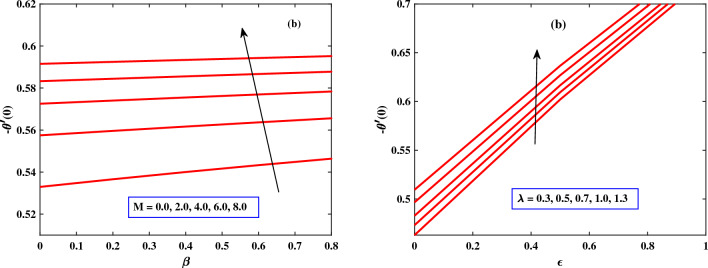
Behaviour of Nusstel number under the influence of *M* and $\lambda$.

**Figure 10:**
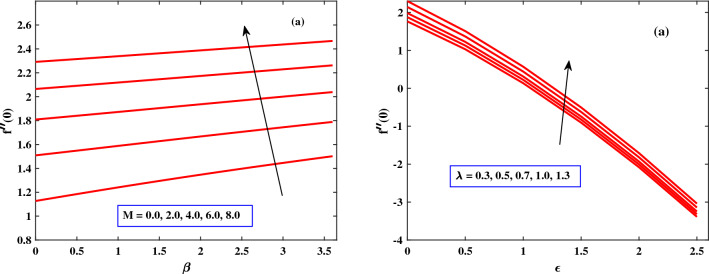
Behaviour of skin friction under the influence of *M* and $\lambda$.

In Figs. 9 and 10, the behavior of skin friction and Nusselt number are discussed for different parameters. The skin friction and Nusselt number show dynamically increasing behavior due to the increased values of Harman number *M*, and $\lambda$ respectively. The presence of *M* and $\lambda$ can cause the fluid to mix more vigorously, leading to improved heat transfer. This enhanced mixing helps in breaking up boundary layers and increasing the convective heat transfer coefficient, thus increasing the Nusselt number. The increase in skin friction due to the Hartman number and $\lambda$ can cause flow distortion and energy losses in the system. This effect is particularly significant in situations where minimizing energy losses and optimizing flow efficiency are crucial, such as in certain industrial applications, energy generation systems, or aerospace engineering.

## Conclusions

This research aims to explore how magnetized nanofluid and Maxwell fluid flow under the influence of mixed convection with convection and heat source over two-dimensional stretching sheets with activation energy. The Rungaa Kutta method is used to find the mathematical results for the velocity profile, temperature profile, concentration profile, skin friction coefficient, and Nusselt number of the above-mentioned problems. Below is a summary of a few of the most important results.

- The velocity profile $f'(\eta )$ is increasing noticeably with the boosted values of parameters magnetic parameter, Maxwell fluid parameter, thermal mixed convection parameter, velocity ratio parameter, and concentration mixed convection parameter.
- The temperature profile $\theta (\eta )$ is going up when the parameters thermophoresis, Brownian motion, and an external heat source are raised. On the other hand, it goes down when the Maxwell fluid parameter, stretching parameter, Hartman number, and buoyancy parameter are raised.
- Nanoparticle concentration profiles get lower as Brownian motion and chemical reaction parameters go up, and they change shape as boosted values of thermophoresis and activation energy go up.
- The skin friction coefficient and Nusselt number show increasing behavior for increasing valves of Hartman number and buoyancy parameter.

## Future directions

This work serves as a foundation for further exploration in the emerging field of MHD bio-convective three-dimensional rotating flow of nanofluids across stretched surfaces. Possible areas for additional investigation may include:

- **Interdisciplinary collaboration:** Foster interdisciplinary collaboration between researchers in fluid dynamics, bioengineering, and materials science. This collaboration can lead to innovative solutions and a more holistic understanding of the complex interactions within these systems.
- **Exploration of novel materials:** Investigate the use of novel nanomaterials with specific properties to enhance the thermal conductivity and bio-convection effects. This can open avenues for the development of advanced nanofluids with tailored characteristics for specific applications.

## Data Availability

No datasets were generated or analysed during the current study.

## List of symbols

*Nb*: Brownian motion parameter
$$B_o$$: Magnetic field ($$\Omega ^{1/2}\textrm{m}^1 \textrm{s}^{-1/2}\textrm{kg}{1/2}$$)
$$\beta _{t}$$: Thermal expansion ($$\textrm{K}^{-1}$$)
*Q*: Heat source coefficient ($\textrm{Wm}^{-2}\textrm{K}$)
$C_p$: Specific heat coefficient (J)
$$D_T$$: Thermophrosis diffusion $$(\textrm{m}^2\textrm{s}^{-1})$$
$$E_a$$: Activation energy ($$\textrm{J} \textrm{mol}^{-1}$$)
*M*: Hartman number
$\lambda$: Mass free convection parameter
*Nu*: Nusselt number
*Pr*: Prandtl number
$$\beta _c$$: Concentration expansion
$$C_{f}$$: Skin friction
$$\sigma ^{*}$$: Electric conductivity ($$\textrm{S} \textrm{m}^{-1}$$)
$\rho$: Density of fluid ($\textrm{kg} \textrm{m}^{-3}$)
$$\alpha$$: Thermal diffusion ($$\textrm{m}^2 \textrm{s}^{-1}$$)
$$D_B$$: Brownian diffusion ($$\textrm{m}^2 \textrm{s}^{-1}$$)
$\tau$: Specific heat of nanoparticles ($\textrm{JK}^{-1}$)
*k*: Boltzmann constant (k)
$\beta$: Maxwell fluid parameter
*Nr*: Thermal mixed convection parameter
$Q_s$: Heat generation parameter ($\textrm{Wm}^{-1}$)
*Nt*: Thermophoresis parameter
$\epsilon$: Velocity ratio parameter ($\textrm{ms}^{-1}$)
